# Use of healthcare services and expenditure in the US in 2025: The effect of obesity and morbid obesity

**DOI:** 10.1371/journal.pone.0206703

**Published:** 2018-11-07

**Authors:** Michele Cecchini

**Affiliations:** 1 Institute of Global Health Innovation, Imperial College London, London, United Kingdom; 2 Health Division, Organization for Economic Co-operation and Development (OECD), Paris, France; University of Illinois at Chicago, UNITED STATES

## Abstract

**Objective:**

This paper explores the contribution of body-mass index (BMI) categories in shaping past trends of use of healthcare services and associated expenditure in the US and projects results to 2025.

**Methods:**

The study uses Medical Expenditure Panel Survey (MEPS) data for 2000–2012, reweighted on National Health and Nutrition Survey (NHANES) data for 1972–2012 and US Census Bureau data, to carry out projections for up to 2025. A combination of logistic regressions and generalized linear models was used to model use and associated expenditure for the following healthcare services: inpatient care (with/without surgery), office-based care, outpatient-care, drug prescription and home health care. Quantile regressions were used to analyse and project BMI levels.

**Results:**

20.5 million individuals will be severely obese in 2025. Normal-weight and overweight individuals have stable trends in use for many healthcare services. Conversely, use of healthcare services in patients in class II and class III obesity will increase substantially. Total healthcare expenditure increases more quickly in the obese population than in normal-weight individuals.

**Conclusions:**

Class III obesity (BMI≥40 kg/m^2^) significantly affects demand and expenditure for all healthcare services. Careful healthcare service planning and implementing effective policy actions to counteract such trends is crucial to meet future demand.

## Introduction

Despite significant policy efforts to fight unhealthy diets and physical inactivity (e.g. [1,2]), overweight remains a crucial public health challenge for the US. According to the latest estimates, more than 64% of the US adult population has a body mass index (BMI) equal or above 25 kg/m^2^ [3–5]. About half of these individuals are obese (i.e. BMI ≥ 30 kg/m^2^). Recent analyses suggest that the increase in class I obesity (i.e. BMI between 30 and 35 kg/m^2^) may be occurring at smaller rate than prior to 2005 and that, overall, obesity rates may be stabilizing in both adults and youth [6]. Conversely, class III obesity (i.e. a BMI≥40 kg/m^2^) is rising twice as fast as overall obesity (i.e. BMI≥30 kg/m^2^) and shows no sign of plateauing [5,7]. Furthermore, different analyses agree that the growth in obesity prevalence is not going to halt for the next ten to fifteen years [5,8,9].

The adverse healthcare consequences of obesity include, among the others, cardiovascular diseases, diabetes and cancers [10,11]. All these diseases require heavy medicalisation and prompt a frequent use of healthcare services. Obese adults and children are more likely to be prescribed drugs [12,13] and to use healthcare services, including diagnostic services [13–17], emergency room visits [13,15,16] and inpatient care [15,17]. Moreover, when using a healthcare service, obese patients require more resource-intensive treatments [18,19].

The associated impact on total healthcare expenditure is substantial. Finkelstein and colleagues [20] conclude that, between 1998 and 2006, obesity-attributable medical spending increased by 45.5% for inpatient care, 26.9% for non-inpatient care and 80.4% for drug expenditure. By using a different approach, Cawley and Meyerhoefer [21] argue that obesity may have a larger impact and, for some healthcare services, costs may be up to 9 times higher than previously calculated. Obese individuals with chronic physical disabilities would incur in almost double costs compared to obese individuals without chronic physical disabilities [17]. Wang [8] calculates that US medical costs associated with common diseases caused by obesity will increase by 48–66 billion USD per year by 2030.

Previous estimates were particularly useful for policy-makers because they justified the implementation of a broad set of preventive interventions to tackle obesity-related risk factors. However, from a healthcare service perspective, available evidence has important limitations. The most significant is that previous studies focus on obesity but do not specifically look at patients with severe obesity. This is particularly important, as patients with moderate and severe obesity have large differences in healthcare costs [19]. Second, available literature does not provide any insight about how healthcare services should adapt to meet future healthcare needs of obese people. This paper builds on the previous research by addressing both of these problems. In particular, this paper explores the contribution of increasing levels of BMI in shaping past and trends of use of a comprehensive set of healthcare services and associated expenditure in the US and project results to 2025.

## Materials and methods

### Methodological overview

Our analyses are primarily based on the Medical Expenditure Panel Survey (MEPS) [22], a dataset reporting information on use of healthcare services and associated expenditure. MEPS data was reweighted to match historical and projected national population statistics to 2015, 2020 and 2025 by using data from the National Health and Nutrition Examination Survey (NHANES) [23] and from the US Census Bureau (USCB) [24]. NHANES was used to calculate BMI prevalence rates and USCB provided information for population distribution by gender, age and ethnicity. The reweighting procedure was carried out on the basis of 384 small homogeneous population groups defined by gender, age group, ethnicity and BMI. A detailed definition of the categories can be found in the S1 Appendix.

### Data

MEPS is an annual survey collecting data on demography, socio-economic status, healthcare utilization and expenditure for the US civilian non-institutionalized population. We used all the waves from 2000 to 2012 and pooled the data for the following six healthcare services: inpatient care (with and without surgery), office-based care, outpatient-care, drug prescription and home health care. Expenditure and charges are expressed in 2010 USD using event-specific product price indexes retrieved from the Office of the Actuary of the Centers for Medicare and Medicaid Services [25].

USCB provides the number of individuals in each population group by gender, age and ethnicity for each year between 2000 and 2025. Reweighting of MEPS data was also extended to data between 2000 and 2012 because discrepancies between MEPS and USCB in the size of the various population groups would have caused spurious inconsistencies between historical and projected trends. NHANES is a study designed to assess the health and the nutritional status of the US population. Our analysis uses all the available waves of NHANES between 1972 and 2012. Prevalence of BMI categories was reweighted on NHANES (rather than using prevalence rates calculated on MEPS) because NHANES covers a longer period (which allows more robust projections) and because NHANES provides the gold standard for BMI estimates in the US.

### Analyses on MEPS

Use of healthcare services was modelled using a two-part model combining a logistic regression to model the probability of use of any specific healthcare service, combined with a generalized linear model (GLM), conditional on having positive access to the healthcare service, to model the average number of use of healthcare services [26,27]. The expenditure specific to each healthcare service is calculated by multiplying the results of the previous two-step model for the average expenditure per access calculated, again, with a GLM model. The Akaike information criterion [28,29] approach was used to select the best fitting combination of link and distribution families in all the GLMs. All the GLMs (but those on use of inpatient services) were calculated by fitting a gamma (Poisson) distribution and a log link. The set of explanatory variables included: gender, age group, ethnicity, level of education, family income, type of insurance coverage, marital status, region of residence, BMI category and year. This approach was shown to be the most suitable to model use of healthcare services and healthcare expenditure [26]. The projections of the use of healthcare services and their associated expenditure were estimated on the fitted models by setting the value of the variables as follows: gender, age group, ethnicity and BMI category were set at the values calculated on NHANES and USCB; all the other covariates, with the exception of the variable year, were set at their values as in MEPS for the period 2000–2012 and at the value they had in 2012 for the projections; the variable year was set at the relevant year (e.g. 2008, 2015, etc.) to account for any underlying trend (e.g. changes in clinical practice).

### Calculation of the weights

For each year, resampling weights are calculated in two steps. In a first step USCB data was used to calculate the relative prevalence of each population group defined by gender, age-group and ethnicity. AS USCB provides both historical and projected population estimates, no further manipulation was necessary. In a second step, NHANES data was used to further split these groups by six levels of BMI for adults [4] and three for children [30]. Waves between 2000 and 2012 provided the prevalence of each population group by BMI level for the relevant period. Projected prevalence rates were instead calculated by using a quantile regression (QR) approach on all the waves between 1972 and 2012. QR was selected because it is particularly efficient when individual quantiles in the distribution of an outcome (i.e. BMI) behave differently along a given dimension (i.e. time). Additionally, QR does not impose any constraint on the future distribution of the outcome variable allowing for higher flexibility in projections and for a non-linear extrapolation of prevalence rates. Thus, it can take into account slowdowns or rapidly increasing trends in specific population sub-groups. All the models include as explanatory variables the same set of variables used on MEPS; in addition the models include a squared version of the variable year as well as interaction terms between year and age group and between gender and education level/socio-economic status (additional information can be found in the S1 Appendix). BMI projections were estimated by setting the time period to the forecasted year (e.g. 2020) while maintaining all the other covariates at the most recent year for which data is available.

## Results

### The US population in 2000 and in 2025: Epidemiologic and socio-demographic developments

Between 2000 and 2025, the US population will go through significant epidemiologic and socio-demographic changes (Table 1). 12.7% of US population was aged 65 or more in 2000. This percentage will increase by about 50% (+5.8% in absolute terms) and people aged 65 or more will become 18.5% of the US population in 2025. The prevalence of white non-hispanic people is also gradually decreasing while the prevalence rate of Hispanics and Asians is expected to grow by more than 50% between 2000 and 2025. Changes in the prevalence of overweight and obesity are also considerable. Almost 64% of the US population will be either overweight or obese in 2025, compared to about 54% in 2000. Severe obesity (i.e. class III obesity) shows particularly high growth rates, rising from about 3.1% of the population in 2000 to 4.5% in 2012 (i.e. +44%), to 6.1% in 2025 (i.e. +35% compared to 2012).

**Table 1:** Key socio-demographic characteristics; prevalence rate in the US population, 2000–2025.

	historic data				projections		
	2000	2004	2008	2012	2015	2020	2025
Population (million)	275.31	285.27	295.01	304.76	312.27	324.93	337.81
Gender							
Males	0.489	0.489	0.489	0.489	0.489	0.489	0.488
Females	0.511	0.511	0.511	0.511	0.511	0.511	0.512
Age							
0–44	0.651	0.630	0.610	0.597	0.591	0.586	0.582
45–64	0.222	0.244	0.260	0.265	0.262	0.249	0.232
65+	0.127	0.126	0.130	0.138	0.147	0.165	0.185
Ethnicity							
White non-Hispanic	0.714	0.697	0.681	0.666	0.655	0.638	0.620
Black	0.128	0.130	0.132	0.134	0.136	0.138	0.139
Hispanic	0.108	0.118	0.128	0.137	0.144	0.155	0.166
Others	0.050	0.054	0.058	0.062	0.065	0.070	0.075
BMI category^a^							
Underweight	0.016	0.014	0.013	0.010	0.009	0.008	0.010
Normal-weight	0.448	0.418	0.398	0.397	0.387	0.369	0.354
Pre-obesity	0.302	0.304	0.301	0.303	0.304	0.309	0.325
Class I obesity	0.147	0.163	0.174	0.180	0.187	0.193	0.181
Class II obesity	0.055	0.062	0.069	0.064	0.064	0.065	0.069
Class III obesity	0.031	0.039	0.045	0.045	0.049	0.056	0.061
Insurance coverage							
public-only	0.726	0.697	0.663	0.644	0.681	0.673	0.664
Private	0.157	0.180	0.203	0.229	0.192	0.200	0.209
out-of-pocket	0.117	0.123	0.134	0.127	0.127	0.127	0.126

^a^ BMI (Body Mass Index) categories are defined according to WHO thresholds (WHO, 2016) for adults and Cole et al. 2000 for children and teenagers

### Use of healthcare services and expenditure at different levels of obesity

Throughout the studied period, the rate of drug prescriptions for obese people is, on average, 2.4 times higher than for normal-weight people (Table 2). However, individuals in class III obesity show a rate of drug prescription which is, on average 3.6 times higher than normal-weight individuals. Similar patterns can be identified for all the other types of healthcare services included in the study. For instance, compared to normal-weight people, individuals in class III obesity are respectively 2.1, 2.7 and 4.5 times more likely to be admitted to a hospital, to undergo surgery or to use home healthcare services.

**Table 2:** Healthcare service use and (standard error) per 1000 persons by year, category of service and BMI; US population 2000–2025.

BMI^a^		2000		2012		2015		2020		2025	
		Value	SE	Value	SE	Value	SE	Value	SE	Value	SE
Prescriptions	[18.5–25]	6081	156	7223	202	7558	258	8092	331	8875	434
	[25–30]	9539	217	11285	263	11647	332	12081	415	12485	516
	[30–35]	11693	261	13921	309	14352	388	15542	505	15884	636
	[35–40]	17206	416	20389	497	21692	620	23197	786	25698	1018
	[40+	20723	541	25215	650	26721	795	30623	1027	35382	1355
Inpatient care (no surgery)	[18.5–25]	215	6	198	6	191	7	182	8	175	9
	[25–30]	242	7	224	6	215	7	205	8	195	9
	[30–35]	282	8	261	8	251	9	244	10	235	11
	[35–40]	344	11	322	10	316	11	312	13	312	15
	[40+	426	1	406	15	397	16	396	18	397	20
Inpatient care (surgery)	[18.5–25]	86	4	77	4	74	5	70	5	66	6
	[25–30]	111	5	100	5	97	6	90	6	83	7
	[30–35]	128	6	115	6	111	6	106	7	100	8
	[35–40]	174	9	159	8	160	9	157	11	157	12
	[40+	211	11	199	11	199	13	201	15	205	18
Office-based care	[18.5–25]	4219	103	4308	108	4329	127	4348	150	4403	180
	[25–30]	5181	119	5278	122	5235	143	5144	169	5058	198
	[30–35]	5793	134	5881	140	5847	163	5917	196	5788	232
	[35–40]	7172	180	7308	199	7430	232	7423	273	7672	328
	[40+	7913	235	8195	249	8318	281	8761	331	9181	393
Outpatient care	[18.5–25]	363	27	266	22	249	26	249	26	196	31
	[25–30]	564	36	411	29	382	35	382	35	273	37
	[30–35]	600	41	439	31	405	36	405	36	299	39
	[35–40]	958	84	714	76	699	89	699	89	550	91
	[40+	1013	81	774	67	751	78	751	78	687	93
Home healthcare	[18.5–25]	79	15	75	14	82	22	82	26	89	33
	[25–30]	103	16	97	15	105	23	100	28	95	32
	[30–35]	99	16	93	15	102	24	111	30	115	39
	[35–40]	167	27	170	28	194	42	221	55	261	74
	[40+	273	44	295	49	348	75	408	97	506	130

The table reports the number of times that a given healthcare service was used per 1000 persons; prescriptions refers to the number of drug prescriptions; SE is standard error

^a^ BMI (Body Mass Index) categories are defined according to WHO thresholds (WHO 2016) for adults and Cole et al. 2000 for children and teenagers.

Obese and severe obese individuals use a significantly higher share of healthcare resources. Normal-weight and overweight people show decreasing or substantially stable trends of use of healthcare services (drug prescriptions excluded). The opposite is true for obese people and, in particular, individuals in class III obesity. In this group, for example, use of home healthcare services is expected to increase from 273 (confidence interval, ci: 187–359) episodes per thousand people in 2000 to 295 (ci: 199–391) episodes per thousand people in 2012 (+8%) to 506 (ci: 251–761) episodes per thousand people in 2025 (+72% compared to 2012). Similarly, the average number of drug prescriptions increased from an average of 20.7 (ci: 19.7–21.8) prescriptions per severe obese patient in 2000 to 25.2 (ci: 23.9–26.5) in 2012 (+22%) to 35.4 (ci: 32.7–38.0) in 2025 (+40% compared to 2012).

As shown in Table 3, obesity is also associated with higher healthcare service-specific expenditure and total expenditure (calculated as the sum of the expenditure for all the healthcare services used by a patient). For example, healthcare expenditure for drug prescriptions and inpatient care with or without surgery is twice as high in obese patients as it is in normal-weight individuals. Similarly, total healthcare expenditure for individuals in class II and class III obesity is, respectively, 2.2 and 2.5 times higher than in normal-weight individuals.

**Table 3:** Healthcare expenditure and (standard error) per capita by year, category of service and BMI; US population 2000–2025.

BMI^a^		2000		2012		2015		2020		2025	
		Value	SE	Value	SE	Value	SE	Value	SE	Value	SE
Prescriptions	[18.5–25]	383	22	641	59	758	88	993	147	1334	237
	[25–30]	601	34	999	58	1166	92	1480	159	1877	250
	[30–35]	717	45	1206	65	1406	103	1862	184	2304	288
	[35–40]	1004	53	1677	96	2016	151	2629	269	3529	446
	[40+	1202	65	2054	121	2463	187	3451	342	4855	585
Inpatient care (no surgery)	[18.5–25]	834	61	939	71	1026	88	1183	115	1395	152
	[25–30]	1145	75	1294	85	1410	106	1604	135	1820	170
	[30–35]	1278	90	1458	97	1594	119	1883	154	2123	196
	[35–40]	1819	136	2085	151	2352	186	2794	241	3367	317
	[40+	2273	181	2680	218	3024	268	3734	349	4670	468
Inpatient care (surgery)	[18.5–25]	735	54	801	61	866	77	961	96	1092	123
	[25–30]	1033	67	1130	76	1218	96	1330	117	1444	142
	[30–35]	1142	78	1255	87	1354	107	1537	134	1663	163
	[35–40]	1627	122	1804	135	2020	167	2316	207	2681	260
	[40+	1977	165	2260	192	2523	234	3001	291	3618	372
Office-based care	[18.5–25]	520	19	886	35	1047	47	1385	70	1848	106
	[25–30]	674	24	1145	40	1341	53	1747	79	2282	115
	[30–35]	758	28	1285	51	1514	66	2034	98	2611	140
	[35–40]	957	37	1620	67	1944	88	2548	128	3457	189
	[40+	1028	54	1773	95	2123	119	2945	170	4039	246
Outpatient care	[18.5–25]	338	31	328	32	334	38	334	38	373	56
	[25–30]	529	41	514	44	520	52	520	52	556	73
	[30–35]	569	47	561	48	569	56	569	56	604	77
	[35–40]	922	94	899	107	927	121	927	121	1005	154
	[40+	942	94	923	99	950	111	950	111	1142	157
Home healthcare	[18.5–25]	79	20	100	25	124	43	151	60	200	86
	[25–30]	101	21	127	26	155	45	181	61	209	80
	[30–35]	105	23	132	28	165	49	219	73	278	104
	[35–40]	149	32	207	44	267	73	374	111	537	172
	[40+	244	52	354	77	474	132	681	194	1039	301
Total healthcare expenditure	[18.5–25]	2890	16	3695	20	4156	27	5024	39	6243	57
	[25–30]	4083	20	5210	24	5810	32	6879	44	8188	62
	[30–35]	4568	23	5896	27	6603	36	8139	51	9583	72
	[35–40]	6479	36	8292	43	9527	56	11613	78	14576	113
	[40+	7666	47	10043	59	11557	76	14858	106	19363	156

The table reports the average healthcare expenditure per capita; prescriptions refers to the average expenditure for drug prescriptions; SE is standard error

^a^ BMI (Body Mass Index) categories are defined according to WHO thresholds (WHO 2016) for adults and Cole et al. 2000 for children and teenagers.

The expenditure of the majority of healthcare services is expected to grow by about 1.5 to 3.5 times in the period 2000–2025 with about a third of this increase taking place between 2000 and 2012. Healthcare expenditure in obese individuals tends to grow more than in normal-weight individuals. For example, the average expenditure per episode of inpatient care with surgery for a normal-weight patient increases from 735 (ci: 629–841) USD in 2000 to 1,092 (ci: 851–1,333) USD in 2025 (i.e. +49%) while, in the same period, the average expenditure for a class III obese patient increases from 1,977 (ci: 1,654–2,300) USD to 3,618 (ci: 2889–4347) USD (i.e. +83%). Average total healthcare expenditure in 2025 will be more than twice as high as in 2000. In 2025, normal-weight individuals and individuals in class II and class III obesity show an average total healthcare expenditure which is respectively 69%, 76% and 93% higher than in 2012.

### The effect of obesity on the use of healthcare services at the population level

**Fig 1:**
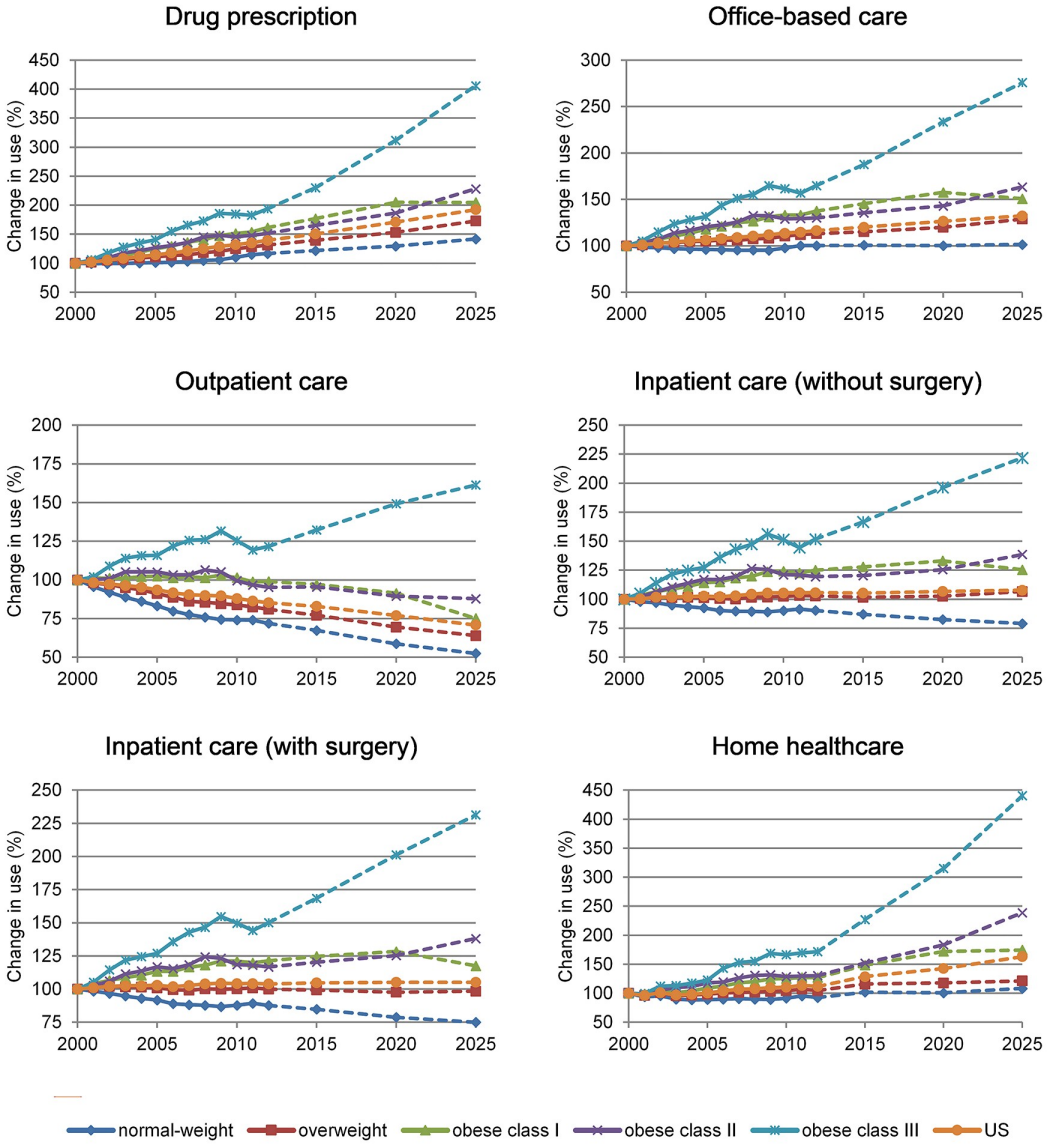
Change in use of healthcare services (%) by category of service and BMI; US population 2000–2025. Note: continuous lines represent historical trends; dotted lines represent projections; BMI (Body Mass Index) categories are defined according to WHO thresholds (WHO 2016) for adults and Cole et al. 2000 for children and teenagers; 2000 is base year equal to 100; additional results in the S1 Fig.

**Fig 2:**
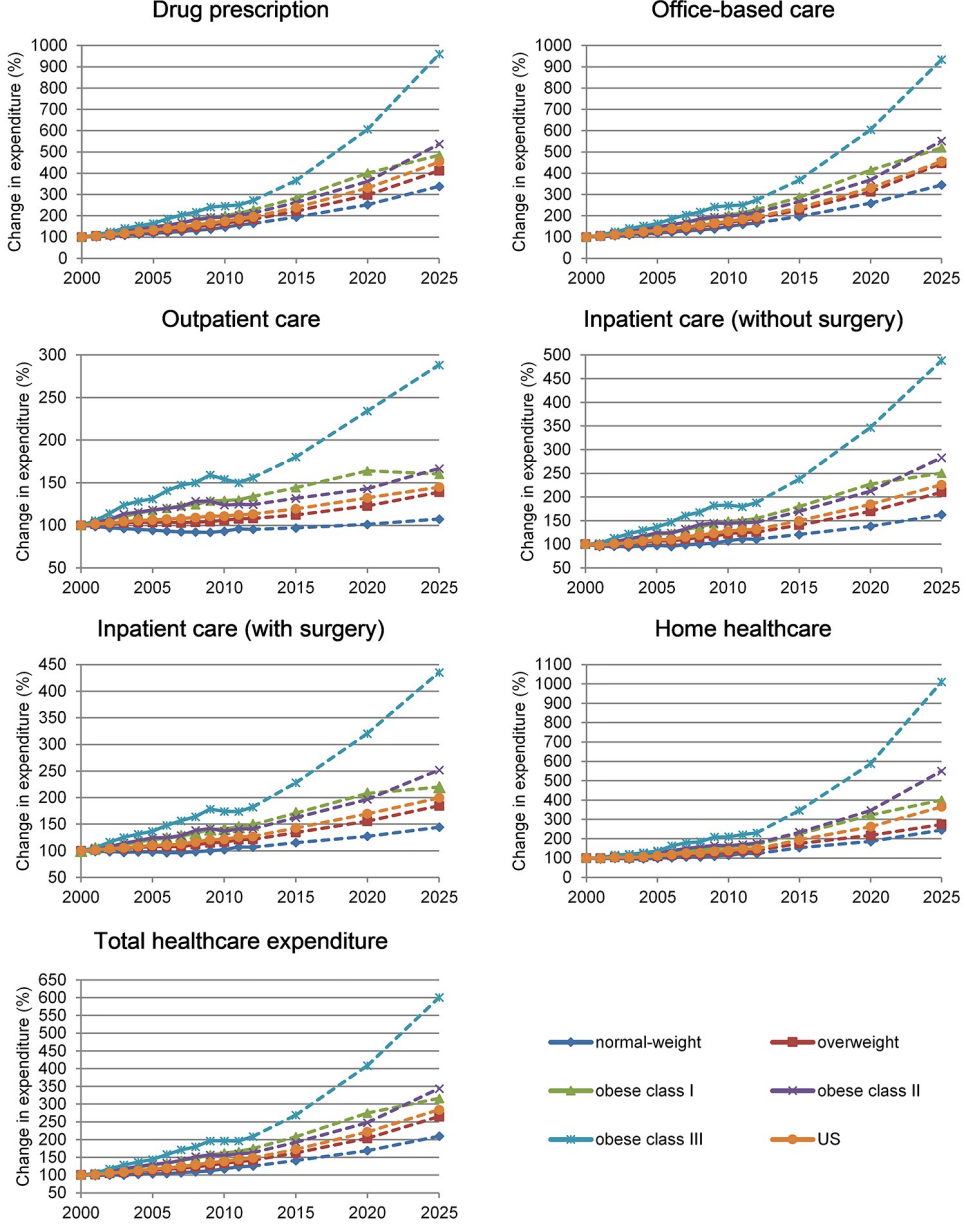
Change in healthcare expenditure (%) by category of service and BMI; US population 2000–2025. Note: continuous lines represent historical trends; dotted lines represent projections; BMI (Body Mass Index) categories are defined according to WHO thresholds (WHO 2016) for adults and Cole et al. 2000 for children and teenagers; 2000 is base year equal to 100; additional results in the S2 Fig.

Figs 1 and 2 show the expected change in use of healthcare services and healthcare expenditure at the population level by BMI category, taking 2000 as the base year. These two figures are calculated by taking into account changes in use of healthcare services and expenditure at the individual level as presented in Tables 2 and 3 and the number of individuals in each BMI category (Table 1). At the population level, use of all healthcare services, but outpatient care, is expected to increase (Fig 1 and additional results in the S1 Fig). Drug prescriptions and use of home healthcare services are the healthcare services increasing the most. Inpatient care with or without surgery is projected to increase, but at a smaller rate.

After scaling-up results presented in Table 2 to the population level (i.e. by taking into account the number of individuals in different BMI categories and its evolution over time), normal-weight and overweight individuals show decreasing or stable trends in use of many healthcare services (e.g. outpatient care and inpatient care with or without surgery). Trends in use of healthcare services for individuals in class I obesity showed a sustained increase between 2000 and 2012. Such increase is projected to continue, but at a smaller rate, between 2012 and 2020 and to stabilize (i.e. drug prescriptions and home healthcare services) or even reverse (i.e. all the other healthcare services) after 2020. Conversely, use of healthcare services for individuals in class II and class III obesity increases consistently. This pattern is particularly clear in the case of outpatient care services. In fact, total number of outpatient visits is expected to decrease for all the BMI categories with the exception of individuals in class III obesity whose total number is expected to increase by 60% between 2000 and 2025.

At the population level, real-term total healthcare expenditure (i.e. after adjusting for inflation) increased by about 50% between 2000 and 2012 and is expected to increase by a further 91% between 2012 and 2025. Total healthcare expenditure is expected to increase more quickly in the obese population. For example, total expenditure for individuals in class III obesity increased by about 110% between 2000 and 2012 and is projected to increase by about 190% between 2012 and 2025.

Healthcare service-specific expenditure is also expected to increase. Drug prescription and office based care show the greatest growth (i.e. about 4 times between 2000 and 2025), while outpatient care and inpatient care with surgery show the smallest increase (i.e. 1.5–2 times). Expenditure in class I to class III obese patients consistently grows more quickly than in normal-weight and overweight individuals. Real-term expenditure for outpatient care in normal-weight individuals is projected to remain virtually stable throughout the studied period.

## Discussion

Obesity has been an important factor in determining recent trends of use of healthcare services and associated expenditure. Our analyses suggest that obesity will also play a significant role in the next decade. Only 1 in 3 people living in the US will not be overweight or obese by 2025 while the number of individuals in class III obesity is expected to grow to 20.5 million people in 2025.

Per capita use of many healthcare services, including inpatient care with and without surgery and outpatient care, is generally decreasing over time for all the individuals independently from their level of BMI. Simultaneously, the number of drug prescriptions and use of home healthcare services has steadily increased suggesting that these healthcare services may have allowed a sharp reduction in the number of hospitalizations. Conversely, the average expenditure per episode has been increasing.

By scaling up these trends at the population levels, our analyses suggest that the US should expect a significant increase in the total number of episodes of care and its associated expenditure. Two crucial drivers of such increase are the growth in the obese population, severely obese individuals in particular, and a higher average expenditure per episode of care. Conversely, decreasing trends in average use of some healthcare services and a levelling-off of the expenditure for overweight individuals, whose expenditure is converging towards the levels of normal-weight individuals help prevent a more significant increase in total healthcare expenditure.

### Policy implications

From a policy perspective a crucial issue is how decision makers can respond to these trends. A sustained emphasis on policies to encourage healthier lifestyles may contribute to further curb obesity rates. Actions to prevent obesity were shown to save up to 8.3 USD in healthcare expenditure for each US resident [26]. About two thirds of these savings would derive from decreased expenditure for inpatient care, followed by a 30% decrease in expenditure for drug prescriptions. However, while prevention has the potential to play a significant role in curbing rising healthcare expenditure, it is unlikely that prevention can reverse such trends on its own. Our analyses suggest that inpatient services are increasingly substituted by pharmaceutical prescriptions and, possibly, by a wider use of other healthcare services. The expenditure for these healthcare services is lower compared to the expenditure for inpatient care. Moreover, there is evidence that substituting inpatient care with drug prescriptions [31] and ambulatory care [32] may reduce total healthcare expenditure without affecting the health of the population. Policy makers, therefore, should be encouraged to put in place new actions aimed at supporting and reinforcing substitution of resource-intensive healthcare services with more efficient alternatives.

Another crucial challenge is adapting healthcare services to meet a changing population with new healthcare needs. Use of healthcare by class II and class III obese people is projected to grow significantly and, inevitably, healthcare facilities will have to adapt to treat a higher number of severely obese patients. This will require investments on designing suitable facilities and space, on proper equipment and on training and implementing new clinical protocols [33]. Some of these changes, particularly those concerning structural renovation and removal of architectural barriers, require substantial time and planning before their implementation. It is therefore crucial that healthcare services start working on required arrangements in the short term.

### Comparison of the results from this study with previous attempts

Our results compare favourably to previous research in the field. Two previous studies [8,34] projected that about 45–50% of the US adult population would be obese in 2030. Finkelstein and colleagues [9] instead predict that, respectively, 42% and 11% of the US would be obese and severely obese in 2030. Our analyses conclude that about 36% of the population aged 15 and over will be obese and 7.6% will be severely obese by 2025. Two main reasons help explain why we report slightly lower obesity rates. First, our projections use a shorter timeframe, i.e. 2025 as opposed to 2030. Second, our projections are based on more recent data and reflect a recent plateauing of obesity which emerged after the publication of previous studies.

Some previous studies have also analysed historical trends in use of healthcare services and expenditure by level of BMI. In line with the literature (e.g. [15,19]) our analyses conclude that patients in different classes of obesity have a use of healthcare services which is between 2 and 3.5 times higher than in normal-weight patients. Few studies have also attempted to project obesity-related healthcare costs. For example, Wang et al. [34] conclude that expenditure attributable to obesity is expected to roughly double every decade between 2000 and 2030. Despite differences in the scope of the two studies, results of our analysis seem to suggest that we should expect a lower increase in obesity-attributable expenditure, mainly due to lower obesity rates than forecasted by the previous study.

### Study limitations and strengths

A number of limitations should be noted. The most important is that MEPS data report lower estimations of healthcare expenditure compared to estimates based on the National Health Expenditure Accounts (NHEA) [35]. The main reason is that NHEA includes spending for people residing in institutions while MEPS does not. NHEA also includes expenses for services that are not included in MEPS as, for example, over the counter medications. Finally, MEPS is largely based on self-reported information and recall errors or imperfect understanding of questions may not correctly reflect reality. MEPS addresses this issue by supplementing individual-reported information with administrative data. However, it cannot be completely excluded that, in some cases (e.g. individuals denying the permission to contact providers), the procedure is not fully successful. Because of all these limitations, presented estimates are likely to be conservative and increase in healthcare expenditure may be higher.

A second factor may affect our analyses by making our results more conservative. Our analysis aims to understand the specific contribution of obesity in shaping future healthcare needs. To better appreciate the effect of BMI, we decided to maintain constant all the other socio-economic characteristics of the US population. Hence, our projections assume that historical trends will continue into the future with no disruptive modification in how healthcare services are delivered.

A third factor could influence our findings. Obese individuals have a lower life expectancy than normal-weight individuals [36] and, in principle, this could affect our population-level projections. However, USCB projections take into account past trends and current levels of health-related conditions including those caused by obesity [37]. Therefore, our projections should be sufficiently robust to capture any potential future reduction in population size due to higher levels of BMI.

This study enriches the literature in a number of aspects. First, this study explores future trends of use of a wide-ranging set of healthcare services including: inpatient care with surgery, inpatient care without surgery, office-based care, outpatient-care, drug prescription and home healthcare. Second, this study does not exclusively focus on total expenditure but investigates the contribution of each healthcare service and its associated expenditure. Third, this paper pinpoints the effect of the different classes of BMI to allow a comparison with other key drivers influencing future levels of healthcare expenditure. Therefore, compared to previous studies, this paper provides policy-makers unique evidence about expected changes in the demand for specific healthcare services. Finally, from a methodological point of view, results of this paper demonstrate that future analyses projecting healthcare expenditure should account for the effects of key epidemiological trends as increasing levels of obesity. Otherwise, results may significantly underestimate the real change in healthcare expenditure.

## Conclusions

This study shows that, between 2000 and 2025, the US population will go through significant epidemiological and socio-demographic changes. Increasing levels of obesity will be a key driver underlying future rises in use of healthcare services and associated expenditure. In particular, obesity will counteract other factors (e.g. changes in delivery of healthcare due to technological innovation and medical advances) that, otherwise, would produce a decrease in the use of inpatient care and other healthcare services. For example, healthcare expenditure for patients with class III obesity will grow from 6% of total healthcare expenditure in 2000 to about 14% in 2025. If no intervention to curb such trends is put in place, total healthcare expenditure per capita in 2025 could be almost three times what it was in 2000.

## Supporting information

S1 Appendix Additional methodological information. (PDF)

S1 Fig Share of use of healthcare services (%) by category of service and BMI; US population 2000–2025. (PDF)

S2 Fig Share of healthcare expenditure (%) by category of service and BMI; US population 2000–2025. (PDF)
