# Supplementary material for: Use of healthcare services and expenditure in the US in 2025: The effect of obesity and morbid obesity
Source: PLoS One. 2018 Nov 7;13(11):e0206703. doi: 10.1371/journal.pone.0206703 (PMC6221341; doi:10.1371/journal.pone.0206703)
Supplement: S1 Appendix — (PDF) [file pone.0206703.s001.pdf]

S1 Appendix. Additional methodological information

This appendix provides additional information on the methodology used to carry out the analyses on the MEPS and the NHANES dataset followed by additional sets of results.

Analyses on MEPS: data and methods

MEPS is an annual survey that collects data on healthcare utilization and expenditure for the US civilian non-institutionalized population. Each year is representative for the US national population. We used all the waves available from 2000 to 2012 and pooled together the data for the following healthcare services: inpatient care with surgery, inpatient care without surgery, office-based care, outpatient-care, drug prescription and home health care. Expenditure and charges are expressed in 2010 USD using event-specific product price indexes retrieved from the Office of the Actuary of the Centers for Medicare and Medicaid Services [1].

Our estimation strategy largely tracks the approach used in previous studies [2] but we have extended the analyses to a more comprehensive group of healthcare services to allow a more detailed stratification of expenditure. The use of healthcare services is calculated as a two-part model combining a logistic regression to model the probability of use of any specific healthcare service, combined with a GLM, conditional on having positive access to healthcare services, to model the average number of accesses [3]. Healthcare service-specific expenditure is calculated by multiplying the results of the previous two-step model for the average expenditure per access calculated, again, with a GLM model. For each model, the set of explanatory variables includes: gender, age group, ethnicity, level of education, socio-economic status expressed in terms of family income, type of insurance coverage, marital status, region of residence, BMI category and year. Categories and thresholds used to define the explanatory variables can be found in table 1. The two regressions modelling the average cost of inpatient care (both with and without surgery) also include as explanatory variable whether the case was admitted to the hospital as an emergency.

We carried out separate analyses for each of the six abovementioned healthcare services. The specification of each model (including the list of covariates), together with the coefficients of the regression, their standard errors and the p-value are reported in tables 4 to 9.

Table 1. Explanatory variables used to carry out the modelling work on the MEPS dataset and their definitions

Variable name	Number of categories	definitions
Gender	2	Males, females
Age	9	0-7, 8-14, 15-24, 25-34, 35-44, 45-54, 55-64, 65-75, 75+
Ethnicity	4	White non-Hispanic, Black, Hispanic, Others
Insurance cov.	3	Public only, private, out-of-pocket
Level of education	4	No degree, high school or less, bachelor degree, master degree or more
Socio-economic status	5	Below poverty line, 1.01 to 1.24 times poverty line (pl), 1.25 to 1.99 times pl, 2.0 to 3.99 times pl, 4.00 or more times pl
Marital status	3	Single, married, separated/divorced/widowed
Region	4	Northeast, Midwest, South, West
BMI adults (kg/m2)	6	0-18.5, 18.51-25,25.1-30, 30.1-35,35.1-40, 40.1+
BMI children	3	Normal-weight, overweight, obese by age as defined by Cole et al. 2000
Year	16	2000, 2001, …, 2011, 2012, 2015, 2020, 2025

Analyses on NHANES: data and methods

NHANES is a study designed to assess the health and the nutritional status of the US population. Compared to other comparable sources, NHANES provide the advantage of combining interviews and physical examinations of nationally representative samples of the US population. This means that height and weight are measured by professionals following a standardized procedure. Our analyses are carried out pooling together all the available waves from 1972 to 2012. The main outcome variable of interest is personal BMI which has been divided into different categories according to international standards. In particular, for adults, we divided personal BMI into the six standard categories, from underweight to class III obesity, as defined by WHO [4]. For children, instead, we used the BMI thresholds derived by Cole and colleagues [5] and divided the population into three categories.

A quantile regression approach [6] has been used to model historic and future prevalence rates for each BMI category. Compared to other approaches to model BMI, the quantile regression approach has the advantage of projecting BMI values rather than prevalence rates and, thus, does not rest on any assumptions, or impose any constraints, on the future distribution of the outcome variable. Explanatory variables included gender, age group, ethnicity, level of education, socio-economic status, marital status and year. The models include all the abovementioned variables as well as year squared and interaction terms between year and age group and between gender and education/socio-economic status. Categories and thresholds used to define the explanatory variables can be found in table 2.

Quantile regression is a semi-parametric regression approach that can be used to model changes in individual quantiles of the distribution of a continuous outcome variable. In this case it was applied to the continuous distributions of levels of BMI. Quantiles are defined with τ. For example, τ=0.75, Qy(0.75|X) is the 75th percentile of the distribution of y (i.e. the BMI) conditional on the values of x. In other words, 75% of the values of y are less than or equal to the specified function of x. QR minimizes the weighted sum of the absolute deviations of the error term. This means that, at any given percentile τ, the weights τ and (1-τ) are applied to positive residuals (i.e. observations above their respective predicted values) and negative residuals (i.e. observations below their respective predicted values). More formally, the same concept may be expressed as follows:

$$\sum_{i=1}^{n} |y_i - x_i'\beta(\tau)| \cdot \left[(\tau)I\big(y_i > x_i'\beta(\tau)\big) + (1-\tau)I(y_i \leq x_i'\beta(\tau))\right]$$

Where y is the dependent variable, X is a matrix of covariates, β is the vector of coefficients depending on τ (i.e. the quantile that is being estimated) and I is an indicator function that takes the value of 1 if the condition in parentheses is true and 0 otherwise.

Regressions were run for each percentile of the BMI distribution between the 1st percentile and the 99th distribution for both adults (age group 3 to age group 9) and children (age group 1 and age group 2).

Table 2. Explanatory variables used to carry out the modelling work on the NHANES dataset and their definitions

Variable name	Number of categories	definitions
Gender	2	Males, females
Age	9	0-7, 8-14, 15-24, 25-34, 35-44, 45-54, 55-64, 65-75, 75+
Ethnicity	4	White non-Hispanic, Black, Hispanic, Others
Level of education	3	Less than 9 years of school, 9 to 12 years of school, more than 12 years of school
Socio-economic status	5	Below poverty line, 1.01 to 1.24 times poverty line (pl), 1.25 to 1.99 times pl, 2.0 to 3.99 times pl, 4.00 or more times pl
Marital status	3	Single, married, separated/divorced/widowed
Year	16	2000, 2001, …, 2011, 2012, 2015, 2020, 2025

Table 3. categories used to link the datasets and their definition

Variable name	Number of categories	definitions
Gender	2	Males, females
Age	9	0-7, 8-14, 15-24, 25-34, 35-44, 45-54, 55-64, 65-75, 75+
Ethnicity	4	White non-Hispanic, Black, Hispanic, Others
Insurance cov.	3	Public only, private, out-of-pocket
BMI adults (kg/m2)	6	0-18.5, 18.51-25,25.1-30, 30.1-35,35.1-40, 40.1+
BMI children	3	Normal-weight, overweight, obese by age as defined by Cole et al. 2000
Year	16	2000, 2001, …, 2011, 2012, 2015, 2020, 2025

References

1. CMS - Office of the Actuary of the Centers for Medicare and Medicaid Services. National Health Expenditures Accounts: Methodology Paper, 2011. Natl Heal Expend Accounts Methodol Pap. 2011; Available: https://www.cms.gov/Research-Statistics-Data-and-Systems/Statistics-Trends-and-Reports/NationalHealthExpendData/Downloads/dsm-11.pdf
2. Cecchini M, Sassi F. Preventing Obesity in the USA: Impact on Health Service Utilization and Costs. Pharmacoeconomics. 2015;33: 765–76. doi:10.1007/s40273-015-0301-z
3. Martin BI, Turner JA, Mirza SK, Lee MJ, Comstock BA, Deyo RA. Trends in health care expenditures, utilization, and health status among US adults with spine problems, 1997-2006. Spine (Phila Pa 1976). 2009;34: 2077–84. doi:10.1097/BRS.0b013e3181b1fad1
4. WHO. BMI classification [Internet]. [cited 21 Apr 2018]. Available: http://apps.who.int/bmi/index.jsp?introPage=intro_3.html
5. Cole TJ, Bellizzi MC, Flegal KM, Dietz WH. Establishing a standard definition for child overweight and obesity worldwide: international survey. BMJ. 2000;320: 1240–3. Available: http://www.ncbi.nlm.nih.gov/pubmed/10797032
6. Ruhm C. Current and Future Prevalence of Obesity and Severe Obesity in the United States [Internet]. Cambridge, MA; 2007 Jun. doi:10.3386/w13181
