# Supplementary figures and images for: Use of healthcare services and expenditure in the US in 2025: The effect of obesity and morbid obesity
Source: PLoS One. 2018 Nov 7;13(11):e0206703. doi: 10.1371/journal.pone.0206703 (PMC6221341; doi:10.1371/journal.pone.0206703)

**S1 Fig. Share of use of healthcare services (%) by category of service and BMI; US population 2000-2025.**

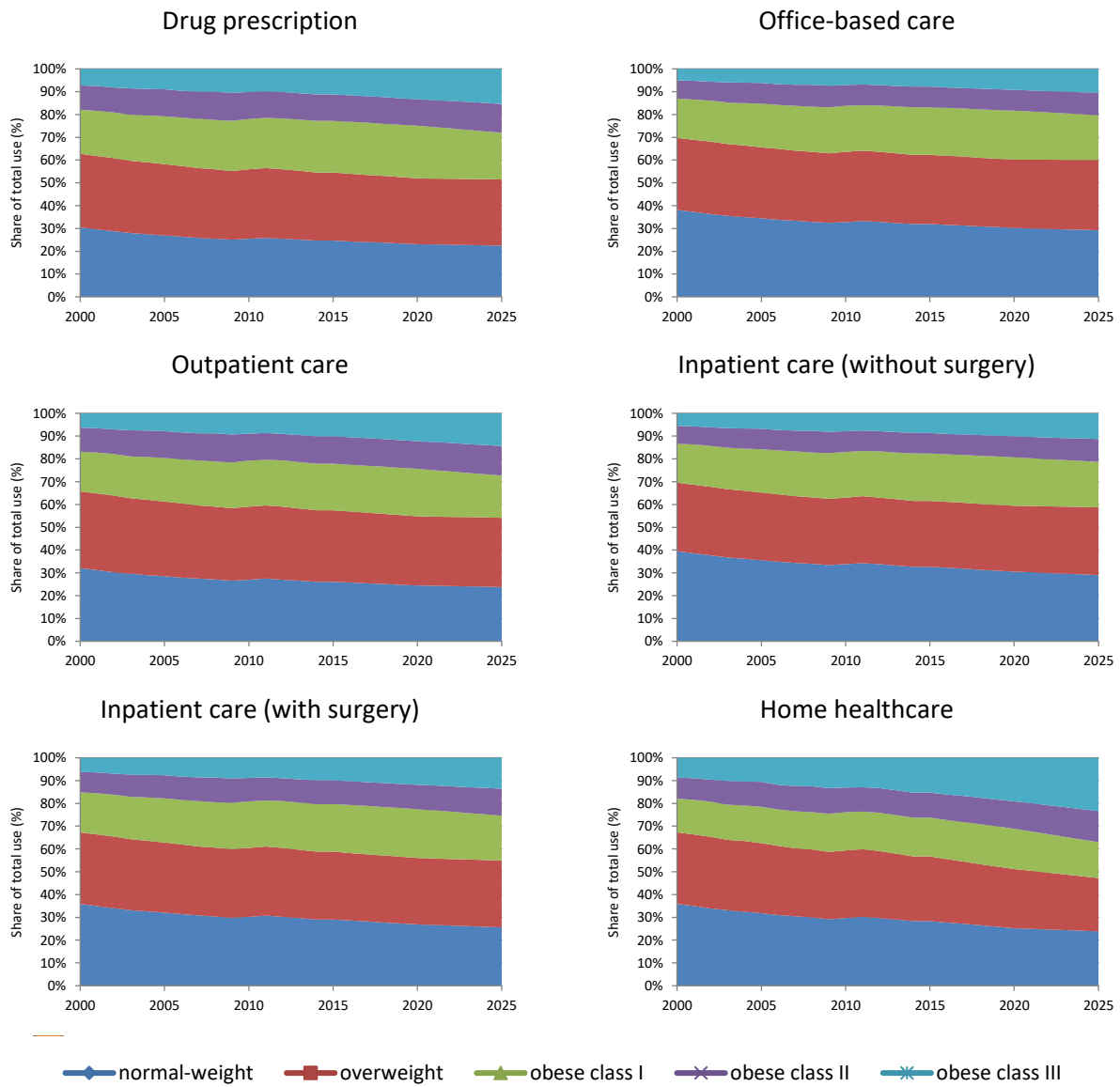

Supplement: S1 Fig — (PDF) [file pone.0206703.s002.pdf]

**S2 Fig. Share of healthcare expenditure (%) by category of service and BMI; US population 2000-2025.**

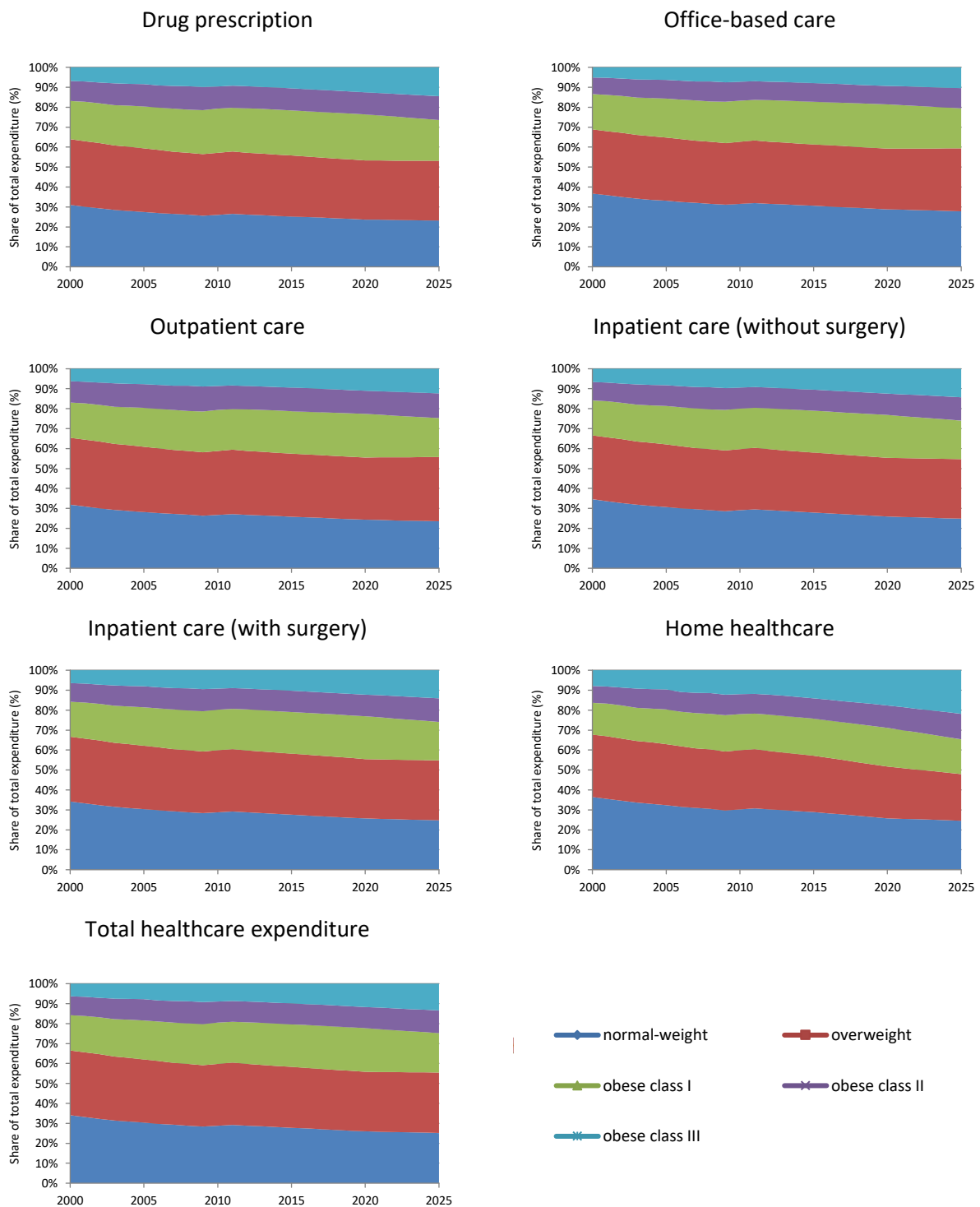

Supplement: S2 Fig — (PDF) [file pone.0206703.s003.pdf]
